# The Effect of Electrical Impedance Matching on the Electromechanical Characteristics of Sandwiched Piezoelectric Ultrasonic Transducers

**DOI:** 10.3390/s17122832

**Published:** 2017-12-06

**Authors:** Yuan Yang, Xiaoyuan Wei, Lei Zhang, Wenqing Yao

**Affiliations:** Department of Electronic Engineering, Xi’an University of Technology, Xi’an 710048, Shaanxi, China; yangyuan@xaut.edu.cn (Y.Y.); leizhang830102@stu.xaut.edu.cn (L.Z.); yaowenqing@stu.xaut.edu.cn (W.Y.)

**Keywords:** sandwiched piezoelectric ultrasonic transducers, electrical impedance matching, effective electro-mechanical coupling coefficient, electro-acoustic power ratio, electro-acoustic gain ratio

## Abstract

For achieving the power maximum transmission, the electrical impedance matching (EIM) for piezoelectric ultrasonic transducers is highly required. In this paper, the effect of EIM networks on the electromechanical characteristics of sandwiched piezoelectric ultrasonic transducers is investigated in time and frequency domains, based on the PSpice model of single sandwiched piezoelectric ultrasonic transducer. The above-mentioned EIM networks include, series capacitance and parallel inductance (I type) and series inductance and parallel capacitance (II type). It is shown that when I and II type EIM networks are used, the resonance and anti-resonance frequencies and the received signal tailing are decreased; II type makes the electro-acoustic power ratio and the signal tailing smaller whereas it makes the electro-acoustic gain ratio larger at resonance frequency. In addition, I type makes the effective electromechanical coupling coefficient increase and II type makes it decrease; II type make the power spectral density at resonance frequency more dramatically increased. Specially, the electro-acoustic power ratio has maximum value near anti-resonance frequency, while the electro-acoustic gain ratio has maximum value near resonance frequency. It can be found that the theoretically analyzed results have good consistency with the measured ones.

## 1. Introduction

Sandwiched piezoelectric ultrasonic transducers are widely applied in areas such as non-destructive testing (NDT) [1], structural health monitoring (SHM) [2,3], energy harvesting [4,5], piezoelectric motors, ultrasonic cleaning, and welding, etc. [6,7,8]. In general, the input electrical impedance of the sandwiched piezoelectric ultrasonic transducers is usually relatively larger. Hence, there is an electrical impedance mismatch between piezoelectric transducers and some interface devices mainly including signal generators, data acquisition devices, which usually have an internal impedance of 50Ω. In addition, on the basis of the Butterworth-Van-Dyke (BVD) model [9], it can be seen that piezoelectric transducers are primarily capacitive when they are operated at resonant frequencies. However, when the input electrical impedance shows capacitive or inductive, the electrical power is obviously less than that for the resistive electrical impedance. Simultaneously, this can also lead to much reactive power. As is well known, the reactive power is quietly harmful to piezoelectric transducers and electrical power sources. Given the above-mentioned considerations, an electrical impedance matching (EIM) network is urgently demanded.

Regarding the investigation of EIM for piezoelectric transducers, the published articles mainly focused on researching the electrical effect, such as bandwidth, the received amplitude, and the power of the acquired signal [9,10,11,12]. In addition, the design of EIM networks for high-frequency transducers has attracted much attention, for example, Lockwood G.R. et al. designed two-element transmission line matching circuits based on network theory [13], Moon J.Y. et al. proposed electrical matching networks based on filter structures [14], and Kim M.G. et al. presented an approach for the design of impedance matching network based on impedance analysis [15]. However, the effect of EIM networks on the electromechanical characteristics for piezoelectric ultrasonic transducers has received little attention.

For investigating the effect of EIM networks on the electromechanical characteristics, a simulated model of the piezoelectric ultrasonic transducer with EIM is highly needed. It is well known that the BVD model is determined from an impedance analysis of piezoelectric transducers, but it cannot be used to directly investigate the electromechanical characteristics of piezoelectric transducers. Based on the Mason’s equivalent circuit model [16] of piezoelectric transducers, the effect of simple EIM networks, such as a shunt inductance, an inductance in series, on the electromechanical characteristics of sandwiched piezoelectric transducers was investigated [17,18], but it is difficult for parameter acquisition and combination with some other module circuits using this method. On the basis of the Leach’s equivalent circuit model [19], Wei X.Y. et al. proposed the PSpice model of single sandwiched piezoelectric ultrasonic transducer in longitudinal vibration [20]; the PSpice model can make us conveniently obtain required parameters and investigate electromechanical characteristics of sandwiched piezoelectric ultrasonic transducers. Piezoelectric transducers are resonant devices, whose resonance frequencies are decided by a lot of factors, such as load variation, ambient temperature variation, and some reactive components,, etc. In order to obtain the optimal operating state, the tracking of the operating frequencies are highly required when the resonance frequencies of piezoelectric ultrasonic transducers are changed [21,22,23].

Given the above-mentioned considerations, based on the established piezoelectric transducer PSpice model [20], the effect of EIM networks on the electromechanical characteristics of sandwiched piezoelectric ultrasonic transducers is investigated in time and frequency domains. Here, the used EIM networks mainly include, series capacitance and parallel inductance (I type) and series inductance and parallel capacitance (II type). In order to further research the effect of EIM networks on the electromechanical characteristics, the pitch-catch setup is built. The dependency of the resonance and anti-resonance frequencies, the electro-acoustic power ratio, the electro-acoustic gain ratio and the effective electromechanical coupling coefficient on EIM networks is derived. It can be found that the theoretically analyzed results are in good agreement with the experimental results.

## 2. Materials and Methods

In this section, the EIM theory for piezoelectric ultrasonic transducers, the used model of single sandwiched piezoelectric ultrasonic transducer, and the parameters calculation of EIM networks, the definition of performance parameters for the single sandwiched piezoelectric ultrasonic transducer, and the pitch-catch setup are illustrated in detail as follows.

### 2.1. The EIM Theory of Piezoelectric Ultrasonic Transducers

**Figure 1:**
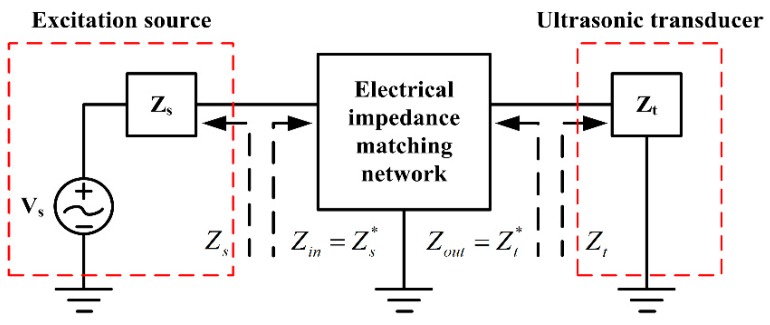
A simplified configuration of an ultrasonic transmitter.

An ultrasonic wave transmitter usually can be simplified, as shown in Figure 1. The EIM network lies in between an excitation source and a piezoelectric ultrasonic transducer. According to the impedance matching theory, the maximum power transmission of ultrasonic transducers can be obtained, when the input and output impedances of an EIM network ($Z_{in}$ and $Z_{out}$) are just equal to the complex conjugates of the electrical impedance of an excitation source ($Z_{s}$) and of a piezoelectric ultrasonic transducer ($Z_{t}$) at resonance frequency, respectively. It is known that the electrical impedance is composed of resistive and reactive components. They can be expressed as

(1) $$\begin{array}{l} Z_{s}(\omega )=R_{s}(\omega )+jX_{s}(\omega )\\ Z_{t}(\omega )=R_{t}(\omega )+jX_{t}(\omega ) \end{array}$$

Here, j represents the imaginary unit and ω is operating angular frequency.

However, as for an optimal electrical impedance matching network, the input and output electrical impedance of the EIM network can be indicated as

(2) $$\begin{array}{l} Z_{in}(\omega )=Z_{s}^{*}(\omega )=R_{s}(\omega )-jX_{s}(\omega )\\ Z_{out}(\omega )=Z_{t}^{*}(\omega )=R_{t}(\omega )-jX_{t}(\omega ) \end{array}$$

Here, $Z_{s}^{*}$ and $Z_{t}^{*}$ are the complex conjugates of $Z_{s}$ and $Z_{t}$, respectively.

### 2.2. The PSpice Model of Single Sandwiched Piezoelectric Ultrasonic Transducer

**Figure 2:**
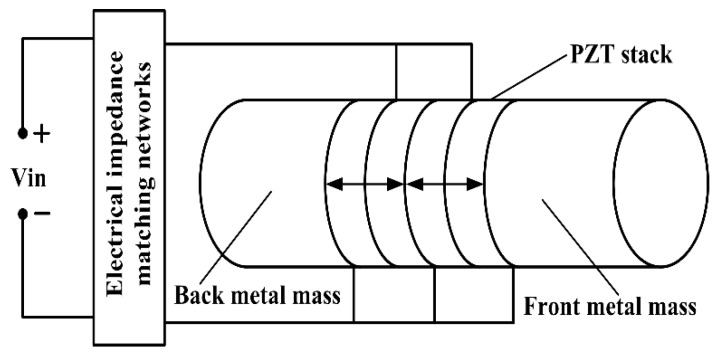
The sandwiched piezoelectric ultrasonic transducer with EIM networks.

The sandwiched piezoelectric ultrasonic transducer with EIM networks is shown in Figure 2. It consists of the thickness polarized piezoelectric ceramic stack, the front and back metal masses, which are clamped together by means of a central prestressed bolt.

**Figure 3:**
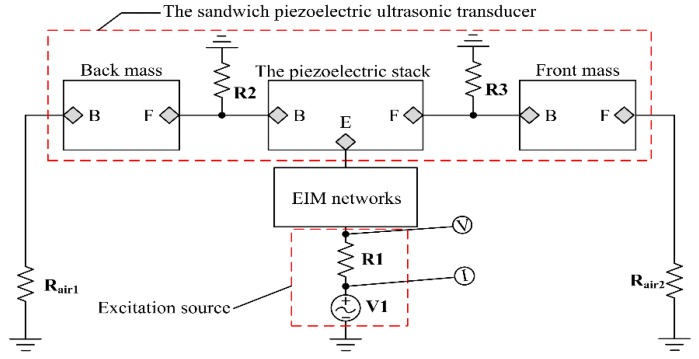
The PSpice model of single sandwiched piezoelectric ultrasonic transducer with EIM networks.

The EIM networks can effectively implement the compensation of the reactance and the tuning of the resistance for single sandwiched piezoelectric ultrasonic transducer. The piezoelectric ceramic stack is composed of four identical piezoelectric rings, which are thickness polarized in opposite directions. The arrow represents the polarized direction of piezoelectric ceramic rings as shown in Figure 2. It should be noted that the piezoelectric ceramic rings located in the piezoelectric ceramic stack are connected together mechanically in series, however they pertain to the parallel relationship in electrical terminals [24]. Specially, to simplify the analysis about the vibration state of the sandwiched piezoelectric ultrasonic transducers from Figure 2, some assumptions must be made as follows: (1) the diameter of the sandwiched piezoelectric ultrasonic transducer must be much less than its longitudinal length; (2) according to the network cascade theory, the piezoelectric ceramic stack can be regarded as a piezoelectric ceramic round stick poled along the axial direction when the thickness of the single piezoelectric ceramic ring is much less than its diameter; and, (3) the displacement and force are continuous on both sides of the connection surface for each component in sandwiched piezoelectric ultrasonic transducers. It should be pointed out that the mechanical boundary conditions of the sandwiched piezoelectric ultrasonic transducers are axially free and radially fixed. But, the radial boundary condition is not fixed in practical application. Then, the PSpice model of single sandwiched piezoelectric ultrasonic transducer with EIM networks based Leach’s equivalent circuit model and transmission line [20] is built, as shown in Figure 3. The resistances $R_{air1}$ and $R_{air2}$ are the air load and have the value $R_{air1}=R_{air2}=0.0263\Omega$ [25]. Also, the resistances $R_{2}$ and $R_{3}$ are used to model the bonded layer and have the value $R_{2}=R_{3}=1.2M\Omega$. Then, the resistance $R_{1}=50\Omega$ is the internal resistance of excitation source $V_{1}$.

**Figure 4:**
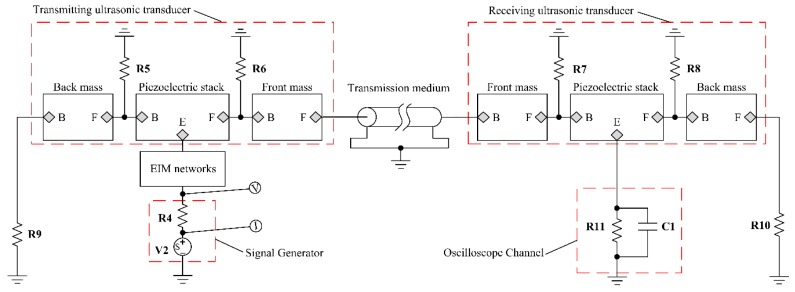
The pitch-catch setup with EIM networks.

On the basis of the PSpice model as shown in Figure 3, the PSpice model of the pitch-catch setup with EIM networks is established as shown in Figure 4. From Figure 4, the pitch-catch setup is composed of excitation source, two sandwiched piezoelectric ultrasonic transducers, and the transmission medium, oscilloscope channel, EIM networks. Besides, it should be pointed that the transmission medium is modeled by the lossless transmission line.

The resistance R11=10MΩ and the capacitance C1=3.9pF are used to represent the oscilloscope channel. Similarly, the resistance R4=50Ω is the internal resistance of excitation source $V_{2}$. Then, the resistances R9 and R10 indicate the air load and have the value R9=R10=0.0263Ω. It should be noted that the materials of the front and back metal masses, the piezoelectric ceramic stack are hard aluminum, steel, and PZT-4, respectively. They are illustrated in detail [20]. Then, the simulated electrical impedance at resonance frequency and the anti-resonance frequency of the piezoelectric ultrasonic transducer and the pitch-catch setup are listed in Table 1.

Here, $f_{s}$, $f_{p}$ are resonance and anti-resonance frequencies, Z is input electrical impedance at resonance frequency. Specially, the simulated electrical impedance at resonance frequency from Table 1 will be used for the parameters calculating of EIM networks in Section 2.4.

### 2.3. The Parameters Definition for the Single Sandwiched Piezoelectric Ultrasonic Transducer and the Pitch-Catch Setup

In this section, the parameters, such as input electrical impedance, reactance and resistance, electro-acoustic power and gain ratio, and vibration velocity ratio, effective electromechanical coupling coefficient are defined.

The input electrical impedance can be expressed as

(3) $$Z_{i}=\frac{V(R1)}{I(R1)}\;\mathrm{and}\;Z_{is}=\frac{V(R4)}{I(R4)}$$

Here, $Z_{i}$ and $Z_{is}$ represent the input electrical impedance of the single sandwiched piezoelectric ultrasonic transducer and the input electrical impedance of the pitch-catch setup, respectively. Then, V(R1) and V(R4) indicate the voltage across resistances R1 and R4, respectively. In addition, I(R1) and I(R4) are the current of through resistances R1 and R4.

From Equation (3), the reactance and resistance can be obtained as

(4) Ri=Re[Zi] and Xi=Im[Zi]

(5) Ris=Re[Zis] and Xis=Im[Zis]

Here, $R_{i}$ and $X_{i}$ are the resistance and reactance of single sandwiched piezoelectric ultrasonic transducer, respectively. Then, $R_{is}$ and $X_{is}$ are the resistance and reactance for the pitch-catch setup, respectively.

From Figure 3, the electro-acoustic power and gain ratio for single sandwiched piezoelectric ultrasonic transducer can be derived as

(6) $$\eta_{P1}=\frac{P_{1}+P_{2}}{P_{i1}}\;\mathrm{and}\;\eta_{G1}=\frac{V_{2}}{V_{1}}$$

where $\eta_{P1}$, $\eta_{G1}$ are the electro-acoustic power and gain ratio, respectively. Then, $P_{1}$, $P_{2}$ represent the consumed powers of resistances $R_{air1}$ and $R_{air2}$ separately while $P_{i1}$ is the input electrical power. $V_{1}$, $V_{2}$ are the excitation voltage and the radiated acoustical pressure from the front metal mass, respectively.

Then, the vibration velocity ratio between the front mass and the back mass is expressed as

(7) $$\eta_{v}=\frac{V_{P-P}(R_{air2})}{V_{P-P}(R_{air1})}$$

Here, $V_{P-P}(R_{air2})$, $V_{P-P}(R_{air1})$ represent the vibration velocities of the front mass and the back mass, respectively.

Similarly, from Figure 4, the electro-acoustic power and gain ratio for the pitch-catch setup can be obtained as

(8) $$\eta_{P2}=\frac{P_{3}+P_{4}+P_{5}}{P_{i2}}\;\mathrm{and}\;\eta_{G2}=\frac{V_{4}}{V_{3}}$$

Here,$\eta_{P2}$, $\eta_{G2}$ are the electro-acoustic power and gain ratio, respectively. Then, $P_{3}$, $P_{4}$ indicate the radiated powers from the front and back metal masses, respectively. $P_{5}$ is the received power, while $P_{i2}$ is the input electrical power. In addition, $V_{3}$ and $V_{4}$ are the excitation voltage and the received voltage.

In addition, the effective electromechanical coupling coefficient $k_{effc}$ can be indicated as [19]:

(9) $$k_{effc}={\left(1-{\left(f_{s}/f_{p}\right)}^{2}\right)}^{1/2}$$

Here, $f_{s}$, $f_{p}$ are resonance and anti-resonance frequencies, respectively. It should be noted that the effective electromechanical coupling coefficient $k_{effc}$ depends on many factors, such as the materials, dimensions and structures, the electrical and mechanical loads.

### 2.4. The Parameters Calculation of EIM Networks

**Figure 5:**
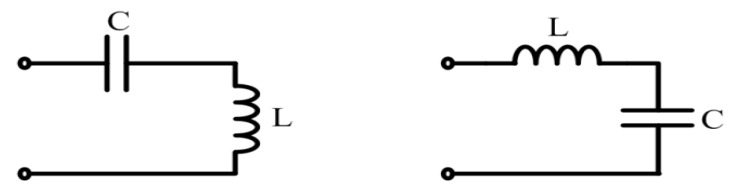
The used EIM networks.

In this section, the parameters calculation of EIM networks for the single sandwiched piezoelectric ultrasonic transducer is illustrated. The specific calculation process mainly has two steps: (1) By using impedance analyzer (PV520A, BEIJING BAND ERA Co., LTD., Beijing, China), the input electrical impedance at resonance frequency is derived; (2) The parameters of EIM networks are conveniently obtained using the Smith chart tool (Smith V3.10 is designed by Prof. Fritz Dellsperger and Michel Band, Bern University of Applied Sciences, Bern, Switzerland.). The EIM networks that are used in this paper, which mainly include I type (series capacitance and parallel inductance) and II type (series inductance and parallel capacitance), as shown in Figure 5.

**Figure 6:**
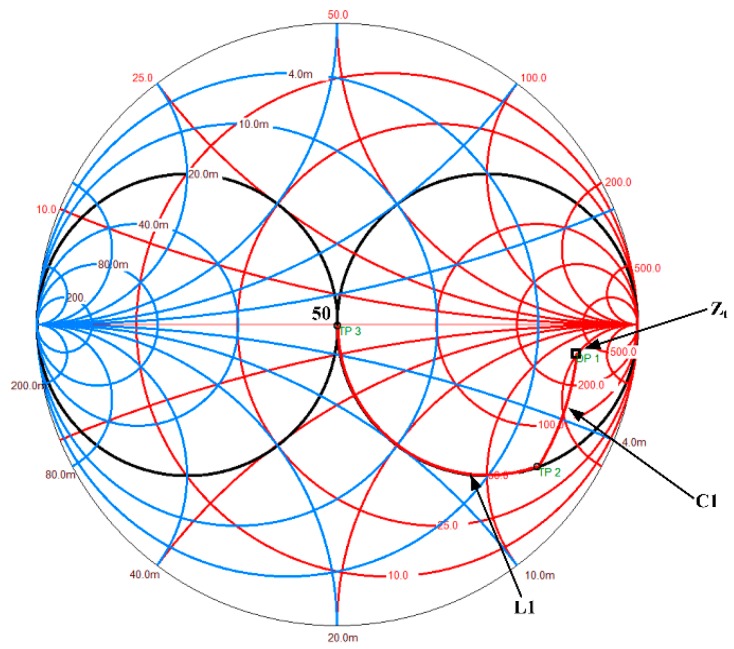
The used EIM networks.

In the case of matching single sandwiched piezoelectric ultrasonic transducer, the load impedance is the input electrical impedance, which is likely to be seen at the far right end of the Smith chart as shown in Figure 6. In general, the source impedance is usually 50Ω, which is located at the center of the Smith chart. The EIM matching network is used, therefore, to move the load impedance to the 50Ω impedance point by means of adding capacitances or inductances in series or in parallel. An inductance in parallel moves the impedance point along the constant conductance curves, while a capacitance in series moves the impedance point along the constant resistance curves. Similarly, a capacitance in parallel moves the impedance point along the constant conductance curves, however, an inductance in series moves the impedance point along the constant resistance curves.

## 3. Results

In this section, the effect of EIM networks on the electromechanical characteristics of sandwiched piezoelectric ultrasonic transducers is investigated in depth in time and frequency domains on the basis of the PSpice models of single sandwiched piezoelectric ultrasonic transducer and the pitch-catch setup, respectively. The detailed analysis process is described as follows.

### 3.1. The AC Analysis for Single Sandwiched Piezoelectric Ultrasonic Transducer

Nowadays, electromechanical impedance technique has been widely used to analyze the electromechanical characteristics of piezoelectric devices and systems [26,27]. Here, this technique is used in analyzing the effect of EIM networks on sandwiched piezoelectric ultrasonic transducers. Based on the above-mentioned PSpice model, as shown in Figure 3, the input electrical impedance is obtained as $Z_{i}=351.362-j186.203$ at resonance frequency $f_{s}=23.309\;\mathrm{kHz}$ by AC analysis. In addition, the calculated parameters of EIM networks are listed in Table 2.

**Figure 7:**
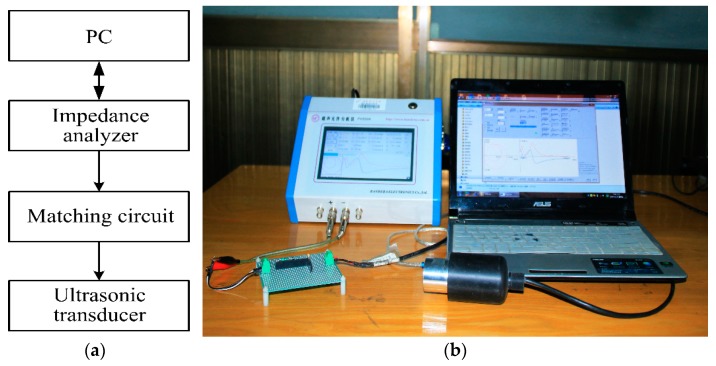
The impedance test of the single sandwiched piezoelectric ultrasonic transducer with EIM network, (**a**) the test platform diagram, (**b**) the test platform picture.

Then, the input electrical impedance and phase, the electro-acoustic power and gain ratio are derived by using AC analysis. For verifying the accuracy of the analysis results, the experimental platform is built which is composed of an impedance analyzer (PV520A made by BEIJING BAND ERA CO., LTD., Beijing, China), single sandwiched piezoelectric ultrasonic transducer with EIM circuits, as shown in Figure 7.

**Figure 8:**
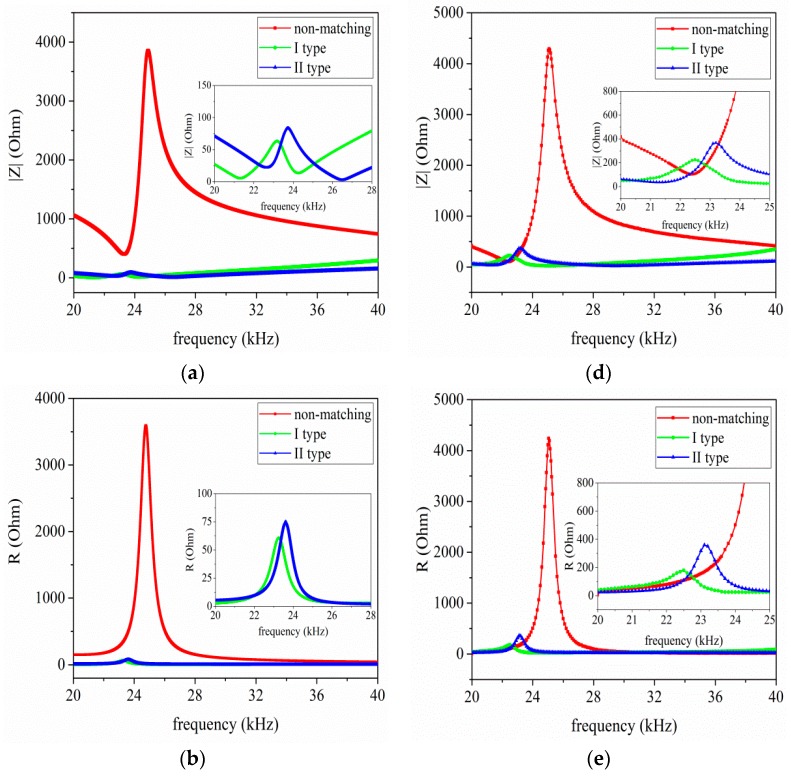

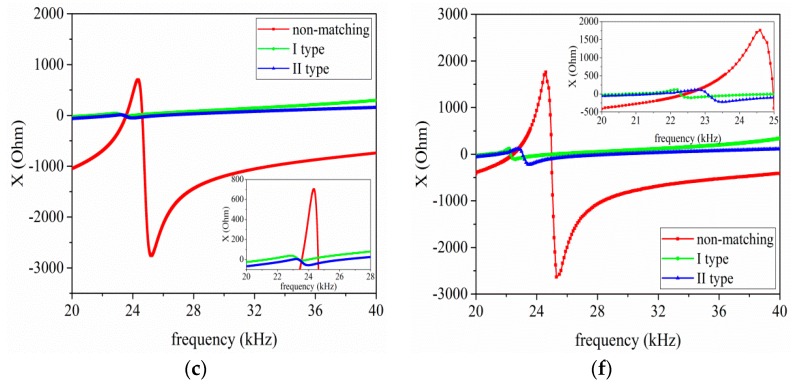
The impedance analysis results, (**a**–**c**) the simulated results and (**d**–**f**) the measured results.

Figure 8a–c describes the simulated results for the relationship between the input electrical impedance, resistance, reactance and the operational frequency. It should be noted that the frequencies corresponding to the minimum and maximum impedance values are resonance and anti-resonance frequencies, respectively. From Figure 8a–c, it can be found that when I and II type EIM networks are connected with the sandwiched piezoelectric ultrasonic transducer, the resonance and anti-resonance frequencies are decreased. Simultaneously, the electrical impedance reaches 50Ω and the reactance closest to zero at resonance frequency. When comparing I type with II type EIM networks, the former makes the resonance and anti-resonance frequencies more substantial decrease. Figure 8d–f illustrates the measured results for the relationship between the input electrical impedance, resistance, reactance, and the operational frequency. It is shown that when I and II type EIM networks are used, the measured impedance, resistance, and reactance are greatly decreased in the whole frequency range of 20 kHz–40 kHz, which has good consistency with the simulated analysis results.

According to Figure 8a,d, the resonance and anti-resonance frequencies and effective electromechanical coupling coefficient are listed in Table 3. In Table 3, $f_{s}$, $f_{p}$, $k_{effc}$ and $f_{ms}$, $f_{mp}$, $k_{meffc}$ are the simulated and measured resonance, anti-resonance frequencies, and the effective electromechanical coupling coefficient, respectively.

From Table 3, it can be seen that when I and II type EIM networks are used, the former makes the effective electromechanical coupling coefficient increase, while the latter makes it decrease. It is shown that the theoretically analyzed results are in good agreement with the measured ones. For the frequency difference of the simulated and measured, the following factors may be able to well explain this problem. Firstly, the used PSpice model of sandwiched piezoelectric ultrasonic transducers is not exactly the same as the manufactured transducers. Secondly, it is well known that the manufactured transducers are different, owing to fabrication error and materials difference, etc. Thirdly, due to the limitation of the values of commercial off-the-shelf components, the calculated values of inductors and capacitors in EIM networks are different from the values of the components used in the experimental platform.

**Figure 9:**
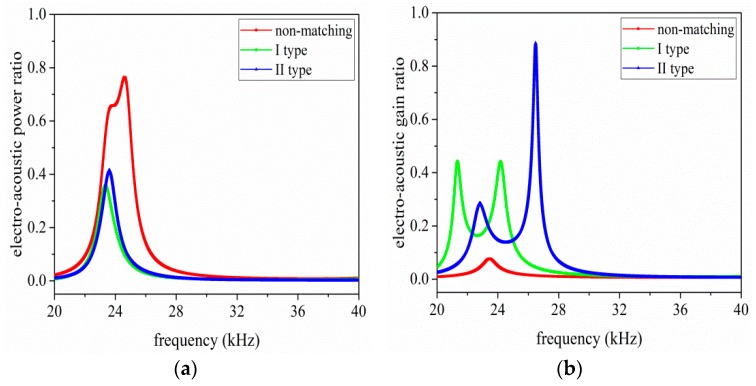
The electro-acoustic characteristics of the single sandwiched piezoelectric ultrasonic transducer with EIM network, (**a**) electro-acoustic power ratio, and (**b**) electro-acoustic gain ratio.

In addition, Figure 9a,b illustrates the relationship between electro-acoustic power and gain ratio and the operational frequency, respectively. From Figure 9a,b, when I and II type EIM networks are used, the electro-acoustic power ratio is decreased, while the electro-acoustic gain ratio is increased at resonance and anti-resonance frequencies. When compared I type with II type EIM networks, it can be found that the latter makes the electro-acoustic power ratio more substantially decrease at resonance frequency, while the former makes it more substantially decrease at anti-resonance frequency; the latter makes the electro-acoustic gain ratio more substantially increase at resonance frequency, and the former makes it more substantial increase at anti-resonance frequency. In summary, it can draw a conclusion that the electro-acoustic power ratio has maximum value near anti-resonance frequency, while the electro-acoustic gain ratio has maximum value near resonance frequency.

### 3.2. The Transient Analysis for Single Sandwiched Piezoelectric Ultrasonic Transducer

**Figure 10:**
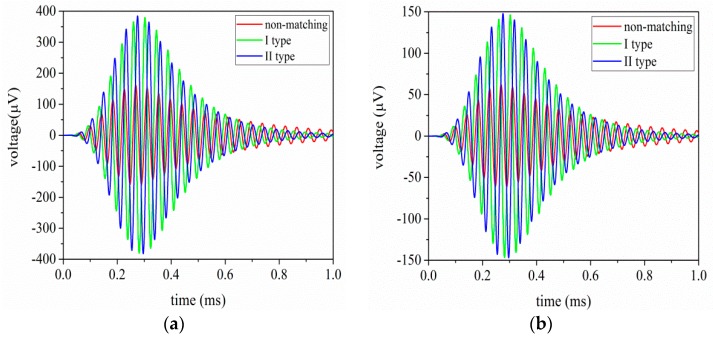
The vibration velocity of the single sandwiched piezoelectric ultrasonic transducer with EIM networks, (**a**) the vibration velocity of the front mass, (**b**) the vibration velocity of the back mass.

In order to research the time domain effect of EIM networks on sandwiched piezoelectric ultrasonic transducers, transient analysis is performed by the PSpice model of single sandwiched piezoelectric ultrasonic transducer, as shown in Figure 3. Figure 10a,b describes the vibration velocities of the front and back masses, respectively. According to Figure 10a,b, the vibration velocity and vibration speed ratio $\eta_{v}$ are listed in Table 4. It can be easily seen that when I and II type EIM networks are connected, the vibration velocities of the front and back masses are increased by more than twice; the vibration velocity ratio is almost unchanged. When comparing I type with II type EIM networks, the latter make the vibration velocities of the front and back masses more substantially increase.

It needs to be pointed that the above-mentioned transient analysis results are derived under no load. The greater the vibration velocity of the front mass is, the greater the ultrasonic wave inserted in the detected objects is.

### 3.3. The AC Analysis for the Pitch-Catch Setup

For further investigation the effect of EIM networks on sandwiched piezoelectric ultrasonic transducers, the AC analysis is performed based on the PSpice model of the pitch-catch setup as shown in Figure 4. The input electrical impedance is derived as $Z_{is}=388.592-j407.495$ at resonance frequency $f_{s}=24.363\;\mathrm{kHz}$ by AC analysis and the detailed parameters of EIM networks are listed in Table 5.

**Figure 11:**
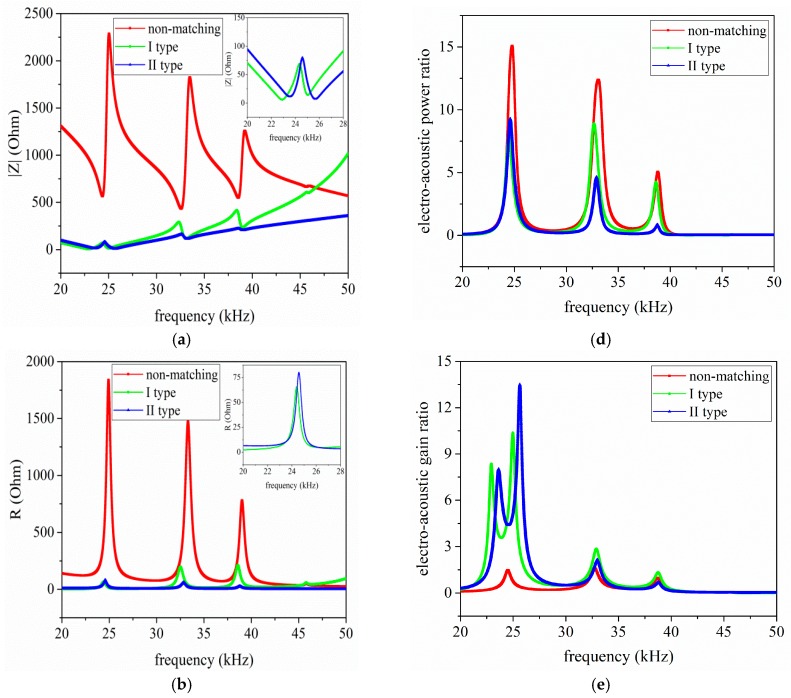

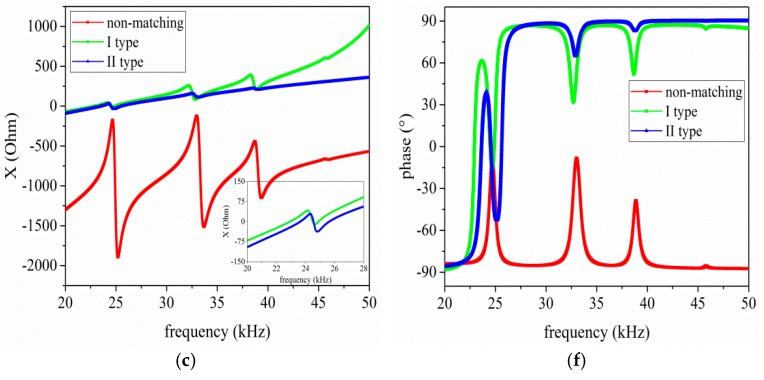
The impedance analysis results of the pitch-catch setup with EIM network, (**a**) electrical impedance; (**b**) resistance; (**c**) reactance; (**d**) electro-acoustic power ratio; (**e**) electro-acoustic gain ratio; and (**f**) electrical impedance phase.

The detailed analysis results are illustrated in below. Figure 11a–c illustrates the relationship between the input electrical impedance, resistance, reactance, and the operational frequency, respectively. In light of Figure 11a–c, it can be seen that the piezoelectric ultrasonic transducers produces multiple resonant modes under having acoustic load. It should be pointed that the analyses below focus on the first resonant mode. When I and II type EIM networks are connected, the resonance and anti-resonance frequencies are decreased.

When comparing I type with II type EIM networks, the former makes the resonance and anti-resonance frequencies more substantially decrease. Then Figure 11d–f describes the variation relationship between electro-acoustic power and gain ratio, the phase of input electrical impedance and the working frequency, respectively.

From Figure 11d,e, when I and II type EIM networks are connected, electro-acoustic power ratio are decreased but electro-acoustic gain ratio are increased at resonance and anti-resonance frequencies. When comparing I type with II type EIM networks, the latter makes electro-acoustic power ratio more substantially decrease at resonance frequency, while the former makes it more substantially decrease at anti-resonance frequency; the latter makes the electro-acoustic gain ratio more substantially increase at resonance frequency, while the former makes it more substantially increase at anti-resonance frequency. Then, according to Figure 11f, it can be found that when II type EIM network is connected, the phase of the input electrical impedance near to zero degree.

### 3.4. The Transient Analysis for the Pitch-Catch Setup

For further investigation regarding the time domain effect of EIM networks on the pitch-catch setup, transient analysis is carried out by the PSpice model, as shown in Figure 4. The pitch-catch setup experimental platform is built so as to verify the accuracy of analysis results. The excitation signal selects 5 cycle sine pulse modulated by Hanning window and the excitation voltage is 20 V.

**Figure 12:**
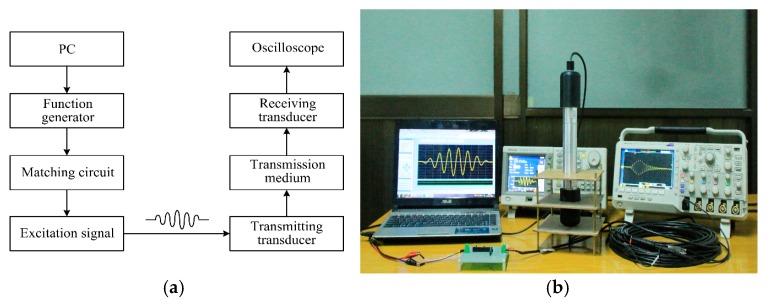
The transient test of the pitch-catch setup with EIM network, (**a**) the test platform diagram; (**b**) the test platform picture.

The experimental platform is primarily composed of two sandwiched piezoelectric ultrasonic transducers, digital oscilloscope, arbitrary waveform generator, personal computer, and some inductors and capacitors, as shown in Figure 12.

**Figure 13:**
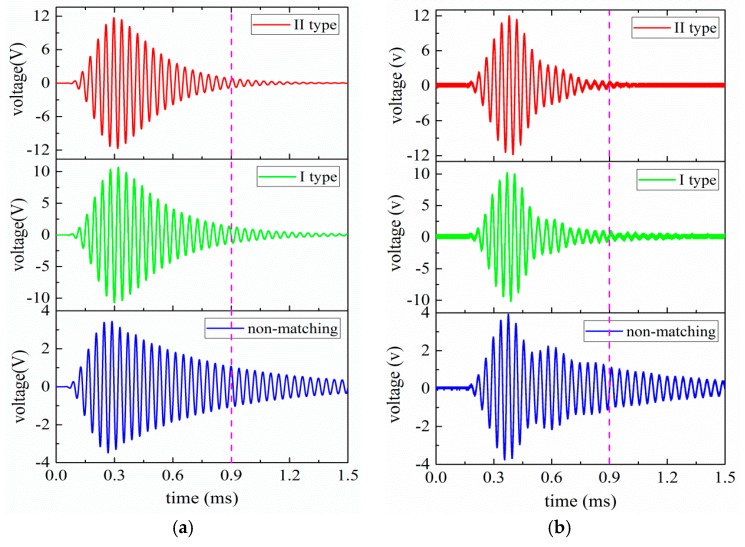
The transient analysis results of the pitch-catch setup with EIM network, (**a**) the simulated results, (**b**) the measured results.

The detailed transient analysis results for the pitch-catch setup are illustrated in Figure 13a,b. From Figure 13a,b, it can be seen that when I and II type EIM networks are used, the oscillation of the received voltage signal is reduced; the latter make the oscillation more substantial decrease. The received voltages of measured and simulated are listed in Table 6. In Table 6, $V_{s}$ and $V_{m}$ are the simulated and the measured voltage values that are derived from the received ultrasonic transducer, respectively and $\Delta_{1}=\left|V_{s}-V_{m}\right|/V_{m}$.

In light of Table 6, when I and II type EIM networks are connected, the received voltage of the sandwiched piezoelectric ultrasonic transducer are increased more than twice. When comparing I type with II type EIM networks, the latter make the received voltage more substantially increase, which has good consistency with the transient analysis results of the single sandwiched piezoelectric ultrasonic transducer. It is shown that the measured results are in good agreement with the simulated ones. But, for the difference in the received voltage in Table 6, there are several factors can well illustrate this problem. Firstly, the used PSpice model of the sandwiched piezoelectric ultrasonic transducer is not exactly the same as the manufactured transducers. Secondly, due to the effect of frequency difference for the used transducer, this can lead to some amplitude error.

**Figure 14:**
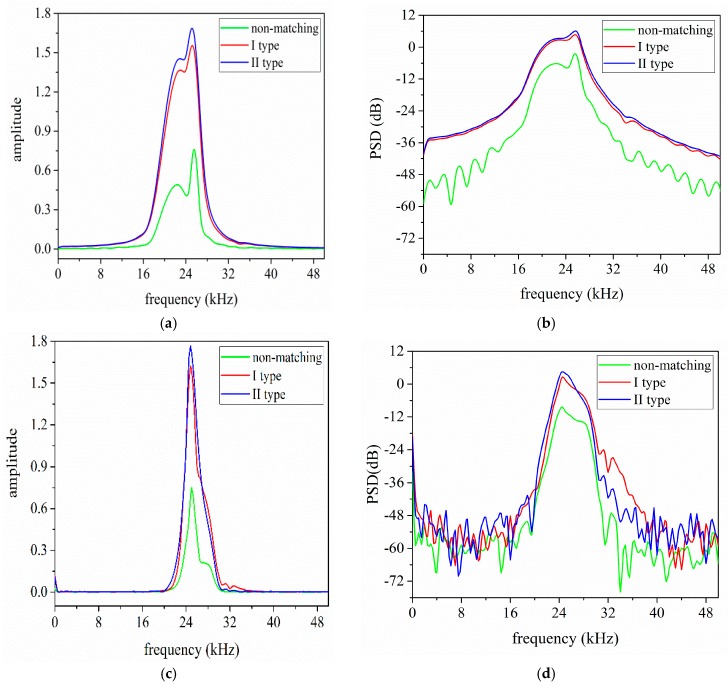
The frequency spectrum analysis results of the received voltage signals, (**a**,**b**) the simulated results; and, (**c**,**d**) the measured results.

For further investigating the effect of EIM networks on the sandwiched piezoelectric ultrasonic transducers, the frequency and power spectrum analysis of the received voltage signals are performed and the analyzed results are shown in Figure 14. Figure 14a,b illustrates the simulated frequency and power spectrum analysis results, and Figure 14c,d describes the measured results. According to Figure 14, it can be concluded that when I and II type EIM networks are connected, the received signal amplitude at resonance frequency in frequency spectrum are increased more than twice; the latter makes the amplitude largely increase.

The specific frequency and power spectrum analysis results for the pitch-catch setup are listed in Table 7. In Table 7, $A_{s}$ and $A_{m}$ are the simulated and measured signal amplitude at resonance frequency in frequency spectrum, respectively. Then, $PSD_{s}$ and $PSD_{m}$ are the simulated and measured power spectral density at resonance frequency, respectively, and $\Delta_{2}=\left|A_{s}-A_{m}\right|/A_{m}$, $\Delta_{3}=\left|PSD_{s}-PSD_{m}\right|/\left|PSD_{m}\right|$. According to Table 7, it can be seen that when I and II type EIM networks are used, the power spectral density are largely increased and the latter makes it more substantially increase.

But, for the difference of the simulated and measured results in Table 7, the following factors can well explain the problem. Firstly, the used PSpice model of single sandwiched piezoelectric ultrasonic transducer is not exactly the same as the manufactured transducers. Secondly, due to the effect of frequency difference for the used transducer, this can lead to some amplitude and power spectral density error.

## 4. Conclusions

By means of the PSpice models of the single sandwiched piezoelectric ultrasonic transducer, the effect of EIM networks on the electromechanical characteristics of the sandwiched piezoelectric ultrasonic transducers is systematically analyzed. To sum up, based on the analysis mentioned above, these conclusions can be drawn as follows:

(1) When I and II type EIM networks are connected with single sandwiched piezoelectric ultrasonic transducer, the resonance and anti-resonance frequencies, the electro-acoustic power ratio, and the received signal tailing are decreased, while the electro-acoustic gain ratio and the power spectral density are greatly increased.
(2) When compared with I and II type EIM networks, the former makes resonance and anti-resonance frequencies more substantially decrease; the former makes the effective electromechanical coupling coefficient increase, while the latter makes it decrease. Then, the latter makes the electro-acoustic power ratio and the received signal tailing more substantially decrease, while it makes the electro-acoustic gain ratio and the power spectral density larger at resonance frequency.
(3) The electro-acoustic power ratio has maximum value near anti-resonance frequency, while the electro-acoustic gain ratio has a maximum value near resonance frequency.

## 5. Discussion

The effect of EIM networks on the electromechanical characteristics for sandwiched piezoelectric ultrasonic transducers is investigated in time and frequency domains by using the PSpice model of the single sandwiched piezoelectric ultrasonic transducer in this paper. For single sandwiched piezoelectric ultrasonic transducer, the effect of EIM networks on the performance parameters is analyzed in detail. The parameters focused attention mainly include the resonance and anti-resonance frequencies, the vibration velocity ratio, the electrical impedance phase, and electro-acoustic power and gain ratio.

When compared with the theory analysis method that is based on the Mason’s equivalent circuit, the proposed analysis method has the following advantages. First, it is extremely easy to obtain the parameters of EIM networks and the performance parameters mentioned above only by AC and transient analysis. Second, the proposed analysis method has great flexibility, which can be very beneficial to optimize the topology of EIM networks. Last but not least, it is certain that the proposed analysis method can provide guidance and basis for choosing the EIM networks. Meanwhile, it should be noted that the equivalent series resistance of the inductances that are used in EIM networks and the prestressed bolt of the sandwiched piezoelectric ultrasonic transducer are neglected. However, in fact, the pre-stress can affect the electromechanical characteristics of the sandwiched piezoelectric ultrasonic transducers. The effect of the pre-stress can be considered in the PSpice model by modifying the related material parameters of the front and back metal masses, the piezoelectric ceramic ring. This will be expected to further investigate in our subsequent work.

## Figures and Tables

**Table 1:** The electrical impedance at resonance and anti-resonance frequencies of the single sandwiched piezoelectric ultrasonic transducer and the pitch-catch setup.

Parameters	$f_{s}(\mathrm{kHz})$	$f_{p}(\mathrm{kHz})$	Z(Ω)
Single transducer	23.309	24.885	351.362-j186.203
Pitch-catch setup	24.363	25.050	388.592-j407.495

**Table 2:** The parameters of EIM networks for single sandwiched piezoelectric ultrasonic transducer.

EIM Networks Type	L(mH)	C(nF)
I type	1.0	47
II type	1.0	33

**Table 3:** Measured and simulated resonance, anti-resonance frequencies and the effective electromechanical coupling coefficient.

EIM Type	$f_{s}(\mathrm{kHz})$	$f_{p}(\mathrm{kHz})$	$f_{ms}(\mathrm{kHz})$	$f_{mp}(\mathrm{kHz})$	$k_{effc}$	$k_{meffc}$
non-matching	23.3	24.9	22.5	25.0	0.35	0.44
I type	21.2	23.2	20.0	22.5	0.41	0.46
II type	22.6	23.9	21.3	23.2	0.31	0.40

**Table 4:** The vibration velocity of the front mass and vibration velocity ratio for single sandwiched piezoelectric ultrasonic transducer.

Parameters	Non-Matching	I Type	II Type
vibration velocity (μV)	322.8	758	769.2
vibration velocity ratio $\eta_{v}$	2.604	2.592	2.603

**Table 5:** The parameters of EIM networks for the pitch-catch setup.

EIM Networks Type	L(mH)	C(nF)
I type	1.2	33
II type	1.5	22

**Table 6:** The received voltages of measured and simulated for the pitch-catch setup.

Parameters	$V_{s}(V)$	$V_{m}(V)$	$\Delta_{1}\%$
non-matching	7.25	7.67	5.5
I type	20.7	21.4	3.4
II type	23.5	23.9	1.7

**Table 7:** The frequency and power spectrum analysis results for the pitch-catch setup.

Parameters	$A_{s}$	$A_{m}$	$PSD_{s}(\mathrm{dB})$	$PSD_{m}(\mathrm{dB})$	$\Delta_{2}\%$	$\Delta_{3}\%$
non-matching	0.43	0.52	−7.91	−8.37	17.3	5.5
I type	1.45	1.53	2.72	2.60	5.2	4.6
II type	1.58	1.67	4.25	4.51	5.4	5.8

## References

[1] Na W.S. (2017). Distinguishing crack damage from debonding damage of glass fiber reinforced polymer plate using a piezoelectric transducer based nondestructive testing method. Comps. Struct..

[2] Song G., Gu H., Mo Y.L., Hsu T.T.C., Dhonde H. (2007). Concrete structural health monitoring using embedded piezoceramic transducers. Smart Mater. Struct..

[3] Wandowski T., Malinowski P.H., Ostachowicz W.M. (2016). Circular sensing networks for guided waves based structural health monitoring. Mech. Syst. Signal Proc..

[4] Pan D., Li Y., Dai F. (2017). The influence of lay-up design on the performance of bi-stable piezoelectric energy harvester. Comps. Struct..

[5] Xu J., Tang J. (2015). Linear stiffness compensation using magnetic effect to improve electro-mechanical coupling for piezoelectric energy harvesting. Sens. Actuators A Phys..

[6] Suzuki T., Ikeda H., Yoshida H. (1999). Megasonic transducer drive utilizing MOSFET DC-to-RF inverter with output power of 600 W at 1 MHz. IEEE Trans. Ind. Electron..

[7] Tuziuti T. (2016). Influence of sonication conditions on the efficiency of ultrasonic cleaning with flowing micrometer-sized air bubbles. Ultrason. Sonochem..

[8] Renshaw T., Wongwiwat K., Sarrantonio A. (2015). Comparison of properties of joints prepared by ultrasonic welding and other means. J. Aircr..

[9] Garcia-Rodriguez M., Garcia-Alvarez J., Yañez Y. (2010). Low cost matching network for ultrasonic transducers. Phys. Procedia.

[10] An J., Zhang S. (2014). A new method of designing electrical impedance matching network for piezoelectric ultrasound transducer. J. Eng. Sci. Technol. Rev..

[11] An J., Song K., Zhang S., Yang J., Cao P. (2014). Design of a broadband electrical impedance matching network for piezoelectric ultrasound transducers based on a genetic algorithm. Sensors.

[12] Huang H., Paramo D. (2011). Broadband electrical impedance matching for piezoelectric ultrasound transducers. IEEE Trans. Ultrason. Ferroelectr. Freq. Control.

[13] Lockwood G.R., Foster F.S. (1994). Modeling and optimization of high-frequency ultrasound transducers. IEEE Trans. Ultrason. Ferroelectr. Freq. Control.

[14] Moon J.Y., Lee J., Jin H.C. (2016). Electrical impedance matching networks based on filter structures for high frequency ultrasound transducers. Sens. Actuators A Phys..

[15] Kim M.G., Yoon S., Kim H.H. (2016). Impedance matching network for high frequency ultrasonic transducer for cellular applications. Ultrasonics.

[16] Mason W.P. (1948). Electromechanical Transducers and Wave Filters.

[17] Lin S.Y. (2017). Study on the Parallel Electric Matching of High Power Piezoelectric Transducers. Acta Acust. United Acust..

[18] Lin S.Y., Xu J. (2017). Effect of the Matching Circuit on the Electromechanical Characteristics of Sandwiched Piezoelectric Transducers. Sensors.

[19] Leach W.M. (1994). Controlled-source analogous circuits and SPICE models for piezoelectric transducers. IEEE Trans. Ultrason. Ferroelectr. Freq. Control.

[20] Wei X.Y., Yang Y., Yao W.Q., Zhang L. (2017). PSpice modeling of a sandwich piezoelectric ceramic ultrasonic transducer in longitudinal vibration. Sensors.

[21] Liu X., Colli-Menchi A.I., Gilbert J. (2015). An automatic resonance tracking scheme with maximum power transfer for piezoelectric transducers. IEEE Trans. Ind. Electron..

[22] Cheng L.C., Kang Y.C., Chen C.L. (2014). A resonance-frequency-tracing method for a current-fed piezoelectric transducer. IEEE Trans. Ind. Electron..

[23] Kuang Y., Jin Y., Cochran S. (2014). Resonance tracking and vibration stabilization for high power ultrasonic transducers. Ultrasonics.

[24] Chen Y.C., Wu L., Chang K.K. (1997). Analysis and simulation of stacked-segment electromechanical transducers with partial electrical excitation by PSpice. Jpn. J. Appl. Phys..

[25] Antlinger H., Beigelbeck R., Clara S. (2016). Investigation and modeling of an acoustoelectric sensor setup for the determination of the longitudinal viscosity. IEEE Trans. Ultrason. Ferroelectr. Freq. Control.

[26] Narayanan A., Subramaniam K.V.L. (2016). Experimental evaluation of load-induced damage in concrete from distributed microcracks to localized cracking on electro-mechanical impedance response of bonded PZT. Constr. Build. Mater..

[27] Yan W., Cai J.B., Chen W.Q. (2011). An electro-mechanical impedance model of a cracked composite beam with adhesively bonded piezoelectric patches. J. Sound Vib..

